# A qualitative investigation into the impact of hemophagocytic lymphohistiocytosis on children and their caregivers

**DOI:** 10.1186/s13023-021-01832-2

**Published:** 2021-05-06

**Authors:** Annabel Nixon, Elina Roddick, Karen Moore, Diane Wild

**Affiliations:** Chilli Consultancy, The Old Fire Station, 2 Salt Lane, Salisbury, SP1 1JS UK

**Keywords:** Hemophagocytic lymphohistiocytosis, Quality of life, Patients, Caregivers, Qualitative research, primary hemophagocytic lymphohistiocytosis, pHLH, Well-being

## Abstract

**Background:**

Primary hemophagocytic lymphohistiocytosis (pHLH) is a rare and life-threatening disorder, which usually occurs during infancy or early childhood and is characterized by abnormal activation of the immune system. However, the burden of pHLH on children and their families has not been previously evaluated. This qualitative study investigated the impact of pHLH and its treatment on the physical, emotional, and social well-being of patients and caregivers in the USA and UK using interviews to provide a comprehensive insight from the perspective of the caregivers and young survivors.

**Results:**

Twenty-one caregivers were enrolled (median [range] age, 41.1 [26–58] years) and represented 20 patients, four from the UK and 16 from the USA. At enrollment, 17 of the 20 patients were alive with a median [range] age of 12.75 [5–31] years at a mean [range] of 7.8 [0.6–11.6] years after diagnosis. In addition, four adult survivors (median [range] age, 23.3 [21–30] years) were also enrolled (total participants n = 25). From noticing initial symptoms to receiving a diagnosis, caregivers reported a mean (range) of 25.9 (0–258) months. pHLH and its treatment had a substantial and long-lasting impact on patients and caregivers, affecting their physical, emotional and social well-being, family relationships, friendships, and ability to work and study. Many of the experiences reported were negative, even after curative treatment, and some participants experienced long-lasting physical and emotional issues. The most noticeable impact of pHLH for patients was on their physical well-being, whereas for caregivers it was emotional well-being. Across all participants there was a sense of isolation due to the illness and its treatment, particularly regarding the patient being immunocompromised and the fear of infection. Areas having a major impact and considered in need of improvement included: delays in diagnosis, lack of patient-specific information on pHLH and a lack of support and understanding about the condition.

**Conclusions:**

pHLH placed a substantial burden on patients and caregivers, which for some were long-lasting. This was compounded by the lack of awareness and understanding of pHLH by healthcare professionals, and a lack of accessible information for those affected by pHLH.

**Supplementary Information:**

The online version contains supplementary material available at 10.1186/s13023-021-01832-2.

## Introduction

Hemophagocytic lymphohistiocytosis (HLH) is a rare and life-threatening disorder characterized by abnormal activation of the immune system, giving rise to hyperinflammation with uncontrolled accumulation of macrophages and lymphocytes [1, 2]. Primary HLH (pHLH) is associated with onset in childhood and an underlying genetic abnormality; secondary HLH is more common and often associated with underlying conditions, such as infection, autoimmune diseases, and malignancies [1–4].

pHLH is considered to be under-recognized and, therefore, its epidemiology is difficult to assess and its world-wide incidence is unknown [5, 6]. However, the incidence of HLH can differ by geographical region [6]. In the USA, the incidence of HLH (primary and secondary) among all children in Wisconsin has been estimated to be 1.5 per 100,000 children per year [7], and in Texas a prevalence of 1.07 per 100,000 children has been reported, which was considered to be an underestimation [8].

Although the majority (70–80%) of pHLH cases begin in the first year of life, pHLH can also present in adolescence and adulthood [6, 9]. Initial clinical manifestations of HLH are similar to severe infections; they are often non-specific and related to a hyperactive immune response, making differential diagnosis challenging for clinicians [1, 5, 9]. A definitive diagnosis is based on a genetic confirmation or the presence of specific clinical signs and laboratory findings [10], which are readily linked to pHLH pathophysiology. Without treatment, symptoms can lead to severe neutropenia, invasive infection, and life-threatening organ damage, resulting in a poor life-expectancy [5, 9]. Conventional therapy aims to control the hyperinflammatory response and lessen the risk of tissue damage [6, 9] so that patients may progress towards allogeneic hematopoietic stem cell transplantation (HSCT).

HSCT utilizes blood- or bone marrow-based stem cells for transplantation; it is the only curative treatment option for patients with pHLH and should be performed in a hospital setting as soon as a suitable donor is available [1, 10]. Therefore, it is important for any treatment to achieve and maintain control of the patient’s condition to allow for HSCT (or a bone marrow transplant (BMT) for stem cells from bone marrow). To achieve this, patients usually receive highly immunosuppressive induction therapy for 8-week (high-dose, daily dexamethasone, plus twice-weekly then weekly etoposide and cyclosporin A) followed by continuation therapy (dexamethasone, etoposide, and cyclosporin A) until a donor is available [10, 11]. Many patients respond to conventional chemoimmunotherapy (71% in remission or alive until HSCT) and go on to HSCT, but for those who do not proceed to HSCT, there are few options available for achieving remission and without HSCT patients are unlikely to survive [11, 12]. For those who do receive HSCT, the 5-year cumulative survival is only 66% [12]. These limitations, along with the significant toxicities associated with current conventional treatments, highlight a need for more effective and safer targeted therapies [13].

Currently, available information focuses on the treatment and management of pHLH, but patient reported outcomes are also important to understand the burden of the illness and its treatment. Therefore, this study investigated the impact of pHLH and its treatment on the health and well-being of patients and their caregivers.

## Results

### Participants

All participants who were successfully screened were included in the study except for one person who withdrew prior to their interview. Participants (n = 25) included the parent/primary caregivers (n = 21) of children with pHLH and young adult survivors (n = 4) who were recruited from across the USA and UK; most resided in the USA (81% of caregivers [n = 17]) and all of the young adults [n = 4]). Twenty-one participants were interviewed face-to-face and four by telephone.

The 21 parents/primary caregivers represented 20 children with pHLH. The mean (range) age of the caregivers was 41.1 (26–58) years with two caregivers having more than one child with HLH. Of the 20 children with pHLH, 17 (85%) were alive at the time of the study and had a mean age (range) of 12.75 (range) of 12.75 (5–31) years. The mean age (range) of the young adult survivors was 23.3 was 23.3 (21–30) years, three were male and one female and all had received stem cell transplantation. The characteristics of the children and the caregivers representing them are summarized in Tables 1 and 2, respectively.

**Table 1:** Patient characteristics: children with pHLH^a^ reported for by caregivers, n = 20

Characteristic	
Sex, n (%)	
Female	8 (40)
Male	12 (60)
Country of residence, n (%)	
UK	4 (20)
USA	16 (80)
Ethnic background, n (%)	
Asian	1 (5)
Black African	1 (5)
Hispanic or Latino	1 (5)
Mixed	2 (10)
White	13 (65)
Other	2 (10)
Surviving children	
Overall, n (%)	17 (85)
Mean age, years (range)	12.75 (5.2–31.05)
Mean time since diagnosis, years (range)	7.8 (0.6–11.6)
Deceased children, n (%)	
Overall	3 (15)
Death due to HLH or treatment	3 (15)
Number of siblings (range)	
Overall number of siblings	0–6
Number diagnosed with HLH	0–2
Number of surviving siblings diagnosed with HLH	0–1
Number of deceased siblings diagnosed with HLH	0–1
Mean age when symptoms first noticed by parents, years (range)	3.95 (0–18)
Mean age at HLH diagnosis, years (range)	6.1 (0–23)
Mean time from first symptoms to confirmed diagnosis, months (range)	25.9 (0–258)
Ever on a waiting list for a HSCT^b^, n (%)	
No	5 (25)
Yes (once)	12 (60)
Yes (more than once)	1 (5)
Yes (number not stated)	2 (10)
Ever had a HSCT^b^, n (%)	
No	2 (10)
Yes, once	14 (70)
Yes, more than once	2 (10)
Yes, number not stated	2 (10)
Treatments received for HLH, n (%)	
Corticosteroids	19 (95)
Chemotherapy	19 (95)
Immunotherapy	18 (90)
HSCT^b^	18 (90)
Other	19 (95)

BMT, bone marrow transplant; HLH, hemophagocytic lymphohistiocytosis; UK, United Kingdom; USA, United States of America

^a^Diagnosis of HLH and need for a BMT used to identify patients with pHLH

^b^Specifically a BMT

**Table 2:** Caregiver characteristics: self-reported

Characteristics	
Number of caregivers interviewed	
Overall, n	21
Face-to-face interview, n (%)	19 (90.5)
Telephone interview, n (%)	2 (9.5)
Sex, n (%)	
Female	17 (81.0)
Male	4 (19.0)
Mean age, years (range)	41.1 (26, 58)
Country of residence, n (%)	
UK	4 (19.0)
USA	17 (81.0)
Ethnic background, n (%)	
Asian	2 (9.5)
Hispanic or Latino	2 (9.5)
Other, Greek European	1 (4.8)
White	15 (71.4)
Other	1 (4.8)
Highest level of education, n (%)	
High school/secondary school	2 (9.5)
Some college or university	5 (23.8)
College or university degree	7 (33.3)
Post graduate qualification	6 (28.6)
Missing	1 (4.8)
Main status, n (%)	
Employed full time	11 (52.4)
Employed part time	2 (9.5)
Other, disability	1 (4.8)
Retired	1 (4.8)
Stay at home	4 (19.0)
Student	2 (9.5)
Number of children that the caregiver was primary caregiving for (range)	0–7
Number of children ever diagnosed with HLH, n (%)	
One	19 (90.5)
Two	1 (4.8)
Three	1 (4.8)
Relationship of caregiver to child/children with HLH diagnosis, n (%)	
Father	4 (19.0)
Mother	16 (76.2)
Sister	1 (4.8)
Number of children deceased attributed to HLH, n (%)	
None	18 (85.7)
One	3 (14.3)
Physical or mental health issues, n (%)	
No	11 (52.4)
Yes	10 (47.6)

HLH, hemophagocytic lymphohistiocytosis; UK, United Kingdom; USA, United States of America

#### Initial symptoms and time to diagnosis

Symptoms of pHLH were first noticed by caregivers when their children were aged between 0 and 18 years (mean age 3.95 years) (Table 1), and between 1 and 19 years (mean age 11.65 years) for the young adult survivors.

Symptoms reported by caregivers ranged widely. The most common clinical symptoms were fevers, organomegaly, jaundice, abnormal blood counts and repeated viral infections, whereas of the subjective symptoms, lethargy and slow recovery from illness were the most reported. These symptoms were often prolonged, lasting for weeks or months.

Although the time from initial symptoms to a diagnosis of pHLH differed substantially for each patient, the mean time was 25.9 months (range from 0 to 258 months) for children (Table 1) and 6 years (range from 0.5 to 21.5 years) for young adult survivors.

#### Delays to diagnosis

One of the main issues raised from the study was the delay encountered before receiving a diagnosis of pHLH (Table 3). The reported reasons for such delays included: an assumption that fevers resulted from viral infections and would resolve in time, incorrect diagnosis of leukemia or cancer, or frequent referrals to different doctors or hospitals (examples of participants’ quotes on delays to diagnosis are provided in Table 3). Notably, many patients became progressively unwell, and some caregivers reported that their child nearly died during the prolonged period towards a diagnosis (Table 3). Many participants perceived a general lack of awareness of pHLH across the medical profession, particularly regarding symptom recognition leading to substantial delays in providing a diagnosis and appropriate referrals. They had concerns about the lack of knowledge of doctors and treatment decisions, and ultimately the difficulties of finding a stem cell donor. This perceived lack of awareness and knowledge of pHLH led to mistrust of healthcare professionals (HCPs) by participants:

“We were very protective of [child with HLH] over who we allowed—‘cause they wanted to study him, you know, we had lots of people coming in and out…. we had a sign on the door that ‘if you can’t say or don’t know what hemophagocytic is, you know, don’t dare step in here’, ‘cause they were doing all kinds of horrible things to him, test-wise…”.

**Table 3:** Delays to diagnosis

Issue	Example quote
Symptom recognition	“Our hematologist was pulling his hair out trying to figure out what was happening, ‘We don’t know what it is’"
Incorrect diagnosis	“The first seven days that we were in the hospital, the doctors still didn’t know what was happening, they thought he just had a bad virus”
Transfer between HCPs	“I requested for him to be transported to [hospital name] and they did. And still, there was like very—a lot of confusion, not knowing what was going on”
Delays leading to severe progression of HLH	“Before he was diagnosed, they ran over 500 tests… I mean, back then, it was even more rare knowing—anybody knowing about this illness … We were so incredibly lucky that his hematologist that we got had just gotten out of his residency and [child with HLH] was very close to dying”
Chance diagnosis	“We were lucky because one of the ER doctors had done a thesis on HLH”

ER, emergency room; HCP, healthcare professional; HLH, hemophagocytic lymphohistiocytosis; HSCT, hematopoietic stem cell transplantation

Note that the words in brackets have been added either to preserve anonymity (following removal of possible identifying information) or for clarity and are not part of the original quote

### Physical well-being

pHLH and its treatment affected the physical well-being of both the patients and caregivers. In patients, the main impact was from pHLH symptoms and the side effects of treatment, whereas in caregivers the burden was mainly due to stress and lack of self-care.

The physical and cognitive development of many patients was delayed, likely as a consequence of the long periods of isolation needed to protect their weak immune systems and the prolonged hospital stays for treatment. As a result, children lost a considerable amount of education time both prior to, and after, diagnosis and some needed additional speech and language therapy. The detrimental effects of treatment on the child’s physical health, which were frequently reported and varied, ranged from hair loss to transplantation issues, such as graft versus host disease, but were considered typical for immunochemotherapy.

Furthermore, changes in the physical appearance of their children after treatment, including after transplantation, were often worrisome for caregivers (Table 4).

**Table 4:** Physical well-being of the patient

Issue highlighted	Example quote
*Before treatment*	
Delayed development	“I knew something was wrong, but I couldn’t put my hand on it either. I mean, it looked—he was developmentally delayed, but when they would test him, they said, ‘Well, he seems okay,’ ‘cause he’d been tested a couple of times, that’s how concerned I was, but again he kept getting sick. We—you know, his neck would just swell and it all seemed lymphatic and sinus related, you know, and then, he had the club feet, so then he finally got through with those shoes and that was fixed and all, and then he slept a lot. He—and then, you know—so, that failure to thrive really lasted, I would say…”
Physical appearance	“…she looked like a scarecrow ‘cause she’d lost so much weight ‘cause she hadn’t been feeling—you know, she’d been sick. She looked, oh, she looked awful. She looked like, you know, a camp survivor, it was terrible…”
Sleep	“He slept. He wasn’t fussy, really, per se, he just was very lethargic. He slept. He didn’t want to wake up to eat”
*Effects of treatment*	
Delayed development	“I would play with him, but even a, let’s say—I don’t know, you know, from the sitting up stage, he was so weak that he had a hard time even playing with toys because, you know, his fine motor—he didn’t walk until he was almost four … And he actually had trauma to his throat when he was intubated. So, they told us he would never talk, so he did all sign language, but now he does talk … I guess four he started getting a voice back. I would say four was the age that he started eating something by mouth. He learned how to walk, he learned how to talk. I would say up until four he pretty much just, kind of, sat there”
Physical appearance: conventional therapy	“So, he was pretty swollen from all the meds [medications] that he was taking. So, like, you know, his cheeks were swollen, and his tummy was swollen”
Physical appearance: HSCT	“So, post-transplant … She added 30 lb to her weight and she’s unrecognizable
Sleep: conventional therapy	“We are weaning him off, according to protocol … cutting down the steroid to zero. And funnily enough, when we cut them down to zero, he slept for the first time, like, a full night … without the—any interruption, without any nightmares, without anything else and woke up, for the first time after nine weeks, with smile on his face”

HSCT, hematopoietic stem cell transplantation

Note that the words in brackets have been added either to preserve anonymity (following removal of possible identifying information) or for clarity and are not part of the original quote

By concentrating on the well-being of their children, caregivers often neglected their own health with marked effects on sleep and physical well-being, for example, poor physical health and exacerbation of existing conditions, including hair loss, migraine, thyroid disorder, worsening epilepsy, and poor recovery from surgical procedures.

## Mortality

Premature death was a key concern following diagnosis, with patients often believing that they would not survive the illness:

“Dad, they said I have HLH. They said I have to do chemotherapy and they said I’m going to be dead in a year, there is no cure”.

Many caregivers had strong negative emotions following diagnosis and reported that the fear of losing their child was one of the worst aspects. Caregivers worried that the death of their child was imminent, and that they may be faced with life-or-death decisions. Several families were actually told that their child was about to die:

“and there’s minimal brain activity and we’re—he’s going to die, you know, you need to—everybody needs to go in and say their goodbyes”.

Some caregivers were advised that pHLH is a terminal illness:

“We have to treat you with chemotherapy like, because otherwise, the mono [mononucleosis] is going to continue to like, kill you and so will the HLH”.

Mortality was assessed as part of physical well-being; however, it had significant overlap with, and had an impact on, emotional well-being.

### Emotional and social well-being

Some participants indicated that the burden of pHLH had a long-lasting effect on the patient (especially young adults), whereas others felt that the patient was not too affected due to their reliance on the caregiver for emotional guidance, for example:

“ …she just looked to us for everything, Am I going to be okay? Is this okay?”

In contrast, caregivers were heavily involved in patient care, both in and out of hospital, and many reported marked effects on their emotional well-being; the stress was compounded by long drives to and from hospital, lack of sleep and isolation. To manage their strong negative feelings and stress many caregivers developed coping strategies, such as positive thinking, support forums and exercise, whereas others resorted to introversion or alcohol use. Patient advocacy forums provided some support; however, participants highlighted the lack of accurate and accessible information about pHLH and treatment options. The challenges of understanding the seriousness of the condition were also considered to have an impact on the well-being of caregivers. However, in some cases, caregivers had a positive influence on the medical care their child received, such as ensuring treatment at a hospital with physicians experienced in pHLH.

The illness and the treatment required were reported to have an impact on the social activities of both patient and caregiver. Before diagnosis, the focus of the caregiver was their child. During treatment their child needed to be isolated due to lowered immunity, frequent hospital visits, and occasionally relocation to expert centers. The effect of this was most pronounced for the caregiver, with several stating that they were entirely consumed by concerns and care for their child with pHLH. This led to a loss of friends and a feeling of isolation, as indicated by one caregiver whose descriptions of the effect of treatment and patient isolation on friendships are below:

“I lost all my friends. I didn’t have no social life at all. It was just revolved around [child with HLH] and trying to look after the kids and cope with what was going on.”

“She was low immune system, so we couldn’t have no friends over at the house.”

These feelings were echoed by many of the caregivers interviewed.

### Relationships

Relationships were often strained within and outside the family. Isolation for treatment and separation from family were key factors.

Caregivers could become overprotective of patients with whom they typically had strong bonds. Often the responsibility of family care rested with one parent or even older siblings, while the other parent focused on the child with pHLH. As a result, family relationships suffered (Table 5). For caregivers and their partners, there was division with regard to the positive or negative impact of HLH on their relationship, i.e. caregivers who received support and had a strengthened relationship with their partners compared with those reporting that pHLH put a strain on their relationship.

**Table 5:** Impact of relationships on patients and caregiver: most commonly highlighted issues

Relationship and issue highlighted	Impact on the patient	Impact on the caregiver
*Patient and caregiver*		
Strong relationship	“Yeah, he’s still close and we’re actually going to see them Saturday, ‘cause he lives in [town name]. But yeah, very close”	“It’s, kind of, you know, it’s probably, you know, created, like, a special bond”
Effect of HLH	“I think it just heightened the relationships that we already had, both for good and for bad”	“I felt like I never really got to enjoy motherhood. She was my first child, you know, I never really developed that normal mother/child relationship. It was, a lot was a sick child/mother relationship…”
Effect of treatment	“When he—we first got admitted, all he did was cry, because he kept saying Daddy and his brother. He wants his brother and his dad and at that time, they told us as much as possible, no siblings under 12 can come to visit. So, physical therapists would come and try to play with him, but no, he was just lying on the bed with me”	“Cause he’s slapped me at some point. He kicked me. He said bad words to me…And then when I said to the doctor that he’s really, his mood is so low, she said, ‘This is steroids, believe me, they are nasty stuff. They make your child be a naughtier child, if you know what I mean? He is not himself’”
*Other children*		
Unable to care for siblings	NA	“It was just, I’d take the kids to school, I’d come home, but I was really like worried about [child with HLH] and that, so my head wasn’t into looking after the kids, ‘cause I couldn’t…”
Negative impact on siblings	“Really bad ‘cause they wasn’t allowed up the hospital, the kids … You’d only seen her for four weeks, hadn’t you? … and then she was took into hospital … She went into hospital on the [date] and never come out until [month], so they never seen her for like, over seven months”	“Whereas [2^nd^ child], yeah, he was—you know, he—it was tough for him. It was tough when—even when he came back to school, we had a little bit of a problems behavior, problems with him and, you know, he was just, you know—he just wanted his family back, you know. He’s used to the full house and, you know, and constant activity and just life wasn’t as it was before. So, he, no, he didn’t take it well for a little while”
Positive impact on siblings: caring and supportive	“She was there … holding her hand. They would snuggle up in bed when [Eldest child] wasn’t feeling well and watch movies. So, it was really all about family for us, 100%”	“So, I had to leave them behind and then, you know, my husband, at the time, stayed with them. But because he worked a lot during the day, she was the one that became responsible, you know, making sure that the seven-year-old would, you know, take a bath, eat, you know, get ready for school, do homework with him, so…”
*Caregiver and partner*		
Support	NA	“Oh, it was so bad. I mean, I was, like, crying every day and again, my husband was the one who was, I would say, the strong one for us, because I would just be crying, and he would be the one telling me, ‘You know, he got through this before, we’re going to get through it again’”
Strain	NA	“I don’t think I could bond with anyone. I just—especially like, my ex-partner, we, sort of like, drifted away. We was just concentrating on the kids and on [child with HLH] and our relationship had basically come to an end”
*Friendships*		
Lost through not understanding	“Her friends did not really keep up with her … And I don’t know if they—you know, her good friends just—they didn’t know how to handle her being so sick or what the problem, what the deal was”	“I lost all my friends. I didn’t have no social life at all. It was just revolved around [child with HLH] and trying to look after the kids and cope with what was going on”

HLH, hemophagocytic lymphohistiocytosis; NA, not applicable

Note that the words in brackets have been added either to preserve anonymity (following removal of possible identifying information) or for clarity and are not part of the original quote

Overall, caregivers felt supported by their extended family; family members and friends provided both emotional and practical support by helping to care for other children in the family, caring for the home, giving financial support, and bringing food to the hospital. Many families in the USA mentioned the positive effect of access to family-centered accommodation close to the hospital (Ronald McDonald House) during the transition from hospital to home, or for a break from hospital.

The nature of pHLH and its treatment, such as isolation or prolonged stays in hospital, affected the patients’ and caregivers’ relationships with school or work. All patients missed a considerable amount of school time; this was especially difficult for older patients and some who experienced difficulties with re-integrating into school, including reports of bullying and social anxiety. Caregivers often stopped working or studying to look after their child and although most returned to work it was often in a different capacity to before their child’s diagnosis. This loss of work and income meant that caregivers experienced financial worries, particularly for participants from the USA who had issues with health insurance. A positive aspect was support from the government or through family/friends fundraising events. Participants were not directly questioned about the financial impact, but many discussed it over the course of the interview, and it was apparent that participants from both the UK and USA had financial concerns, which differed due to different healthcare models. In the USA, the main discussion was around health insurance and community support.

### Life after treatment

Several patients were positive about the improvements to their social life, citing that normality and their physical health returned or was improved following treatment. However, for many patients and caregivers the burden of pHLH persisted beyond HSCT (Table 6). Concerns focused on the possible re-occurrence of pHLH, the challenges of delayed education or achieving their goals, including the need for support from speech and language therapists and the difficulty of reintegrating into school. The long-term or persistent physical impacts of treatment on the patient were also voiced, such as compromised immunity and infections, impact on fertility, inability to be vaccinated, fragile bones, and thyroid deficiency requiring medication. As one caregiver stated:

“And then, thinking about just some of the things that we knew about chemo treatment and how that’s going to impact our little girl, and one—probably the biggest one was, hey, she’s—there’s a chance she’s not going to have kids, and that was—I remember that at the time being just really difficult to be—and it was hard to understand. Like, that seems unacceptable, but then it’s like, ‘Yeah, but we want her to live.’ Like, ‘Oh, okay, yeah, now I see the trade-off. Okay.’ That’s a huge trade-off, but of course, you know, living is ultimately, what you want, you know, and so…”.

**Table 6:** Long-term concerns following treatment

Issue highlighted	Example quote
Anxiety	“Every time she has a sickness, in the back of my head somewhere, HLH is there”
Compromised immune system	‘‘Cause if he got a cold, he’d get it 100 times worse, you know, ‘cause they do, ‘cause their immune system’s not great … [3^rd^ child], I remember he got chickenpox, [child with HLH] caught it. That made him go into hospital, ‘cause they got infected”
Prolonged viral infection and hospitalization	“…about a year past, two years past tran [transplant]—she ended up back in the hospital and we were like—just when you’re like, you know, you breathe a little bit and we’re like, oh, how can this happen two years later?”
Other issues	“…the chemotherapy had killed his thyroid in his neck. I guess, you have a thyroid in your brain as well, but it totally wiped it out. So, he’s on thyroid medication every day…”
Focus on the positives	“So, she is amazing now. So, I mean, she—the more years you put under your belt, the more you relax. So, we didn’t really relax probably—I’d probably say four years, four or five years … She’s now almost off all her meds [medications], it was three years out and she went back to school full-time and, you know, life, sort of, gets back to normal”

Note that the words in brackets have been added either to preserve anonymity (following removal of possible identifying information) or for clarity and are not part of the original quote

pHLH and its treatment had a long-lasting psychological impact on patients and caregivers, including presentation of post-traumatic stress disorder-like symptoms and anxiety about the return of the condition or rejection of the HSCT. Despite surviving, pHLH still places major restrictions on patients’ and caregivers’ lives, such as the need to attend continued outpatient hospital visits for regular post-treatment monitoring, often for months/years after returning home.

## Discussion

Qualitative research has become an important part of medical investigation and knowledge as it can inform clinical practice and patient support [14, 15]. It is uncommon to employ such research in a rare condition such as pHLH; however, information from interviews is valuable to HCPs as it gives a greater understanding of the impact of the whole pHLH journey on patients and caregivers. Furthermore, qualitative studies may supplement the limited published information on the impact of pHLH on the patient and caregiver beyond their medical story.

Treatment decisions influence many different areas of patients’ and caregivers’ lives [16]. However, the burden of pHLH on patients, their caregivers and families has not been previously evaluated, with most studies focusing on its treatment and management. In the current study we have shown that pHLH and its treatment has an enormous and long-lasting impact on patients and their caregivers, across all aspects of life, including their physical well-being. The outcomes from this study provide a comprehensive insight into the overall impact of pHLH from the perspective of the patients’ caregivers and young survivors of pHLH.

One of the key issues for participants was the perceived lack of awareness of pHLH across the medical profession, particularly symptom recognition, leading to delays in diagnosis and referrals. Despite advances in diagnosis and treatment, ongoing education for HCPs is needed so that they can recognize the key features of pHLH, both as typical and atypical presentations [3, 17]. A greater understanding of rare diseases and shared patient journey among individuals with different rare diseases might expedite the diagnosis of pHLH, allowing the correct treatment to be given. A nationwide survey of rare diseases in Germany demonstrated that individuals with rare diseases had shared pre-diagnostic phenomena. In line with our findings these included symptoms that did not improve despite appropriate therapy, symptoms that did not appear to fit together, and/or visiting many doctors [18]. Awareness of these experiences could alert HCPs to the idea of a rare disease. Using pHLH as an example, the implementation of a referral awareness campaign within secondary care hospitals aimed at those patients presenting with febrile illness that does not subside and is not associated with typical etiologies may reduce the time to diagnosis for such patients. In ophthalmology, artificial intelligence techniques have been applied to patient data within electronic health records to improve disease diagnosis, risk assessments and prognosis predictions [19]; however, the application of similar techniques to rare diseases requires further study. Our study suggests that HCPs’ understanding of the whole impact of pHLH (outside of the immediate medical needs) and how to approach and discuss the condition with patients and caregivers needs improvement. However, we acknowledge that some participants in this study were diagnosed up to 10 years ago, and so recent advances may not be apparent in all the shared patient stories.

The persistent nature of pHLH symptoms had a major negative effect on participants; however, this may have been exacerbated by the wide-ranging physical problems that can be caused by conventional chemoimmunotherapy used to treat pHLH. Common side effects associated with chemotherapeutics, such as etoposide, include an increased risk of infection, fatigue, constipation, hair loss, and nausea/vomiting [20], while immunosuppressants, such as cyclosporin A, are associated with neurotoxic side effects, which encompass a wide range of symptoms [21]. In children, the long-term use of corticosteroids, such as dexamethasone, may be associated with toxicities including infections and, for example, weight gain, growth retardation, and Cushingoid features [22]. In the HLH-2004 study [11], the adverse effects of therapy included those on the hepatobiliary system with etoposide treatment, cardiac hypertension with dexamethasone treatment, and the central nervous system with cyclosporin A treatment. Together these toxicities highlight the potential additional burden of conventional treatments on the patient and the need for advances in pHLH therapy.

The impact reported on patients’ physical well-being due to the symptoms and treatment of pHLH may, in part, be exacerbated by the young age of the patients, who may have been too young to understand or articulate their feelings about what is happening to them. Play therapy can be used to reduce anxiety and negative emotions in hospitalized children and may also have a positive effect on caregivers who appreciate the benefits to their children [23]. Given this observation, the provision of play therapists or specialist staff may also be a possible option for children with pHLH to support their understanding of the interventions and treatment required, to express their feelings, which ultimately may reduce their emotional burden and possibly build their confidence in medical personnel. For caregivers, although their physical well-being was adversely affected, the emotional impact of pHLH was particularly marked, especially around the time of diagnosis and while their child was immunocompromised. Therefore, caregivers may benefit from a greater focus on counselling and support for them.

For both patient and caregiver, the sense of isolation as a consequence of illness and treatment was pronounced and affected their emotional well-being, social activities, and relationships. Many caregivers reported strained relationships in the home, with one parent or older siblings left to care for the family, while the caregiver focused on the child with pHLH. Addressing caregiver strain has been noted as an important aspect of maintaining a family-centered approach to patient care [24]. In these circumstances, caregivers may benefit from the availability of accessible tools to aid communication and provide support for caregivers when explaining pHLH to siblings and the extended family, as well as an appropriate level of support from medical staff or informal groups, for those family members who need it. The sense of isolation was compounded by the loss of education or work. Loss of work, as well as treatment costs (US participants), placed a heavy financial burden on caregivers, further straining relationships. In a review of the impact of possible interventions that are available to caregivers of children with chronic and complex medical needs (specifically children with medical complexity), comprehensive insurance coverage and a supportive work environment had a positive impact on well-being; other interventions available included care co-ordination, respite care, telemedicine, peer and emotional support programs, and health and related support [25], some of which may benefit the caregivers of patients with pHLH. As awareness of the condition increases and HLH centers of excellence become established, caregivers may have greater access to accommodation and transport support services. Caregiver stress may be reduced with the right support, and multiple interventions could reduce the burdens of care experienced by families, including time, finances, care needs and access to services [24, 25].

Following their diagnosis many patients in this study were treated at emerging centers of excellence by doctors with knowledge of HLH. As such their post-diagnosis experiences may be better than for patients who have not had access to such care and treatment. At highly specialized centers, multidisciplinary teams are usually central to the management of HLH, and HSCT, and may include, for example, speech and language therapists, psychologists, occupational therapists, physiotherapists, and pharmacists. The mixed experiences of HCP care and understanding encountered by patients and caregivers may have contributed to the general lack of knowledge of pHLH reported by caregivers, which was also reflected in the limited information available to caregivers. Although social media has allowed support groups and centers of excellence to reach out to patients, this study highlighted that more patient-orientated information on pHLH accessible via the internet is required—not only information/literature on symptoms and treatment but also the potential impact on the well-being of patients and caregivers, including potential coping strategies.

Most published studies focus on the pathology and treatment of HLH, and few have evaluated long-term outcomes or the impact on health-related quality of life (HRQoL). In one qualitative study, four caregivers of patients with HLH highlighted the emotional impact and restrictions to children’s lives caused by treatment, which is consistent with our findings for patients [26]. Our study, however, investigated the impact of HLH from before diagnosis through treatment and into the future for both the patient and caregiver in a large sample of participants. Similar to our results, one retrospective study concluded that many children with HLH experience long-term significant cognitive and psychosocial impairments, even after successful HSCT [27], and emphasized the importance of early identification of children with pHLH who are at risk of long-term cognitive and psychosocial difficulties, to optimize support for them.

Currently, there are no HLH-specific patient-reported outcome (PRO) instruments. PRO instruments, such as HRQoL questionnaires, indicate the clinical relevance of standard clinical endpoints and treatment benefits from a patient’s perspective [16, 28]. For long-term conditions, the utilization of relevant PROs gives patients the opportunity to receive high quality care and to discuss the impact of symptoms and treatment on their well-being, which is especially important with targeted treatments. This qualitative study may help researchers to select or develop relevant patient and caregiver reported tools to measure the impact of pHLH and its treatment.

The recruitment of patients with rare diseases into studies is challenging [28] and this may, in part, explain the lack of information on the impact of pHLH and its treatment on patients and caregivers. Recruitment methods, such as identification online, may introduce some bias towards the type of patients recruited [28]. Our methods (online recruitment from advocacy and support groups) successfully recruited an adequate sample of patients and caregivers, although there were inherent limitations. A survival bias for the patients represented in our study (87.5% survival) might have led to an overly positive experience for the participants (for those who receive HSCT, the 5-year cumulative survival is only 66% [12]; only three deceased patients were represented in our study. In addition, there may be some bias towards engaged individuals, those who used support/advocacy groups, but also potentially as a consequence of the number of patients who were treated in centers of excellence and the healthcare that they would have received compared to patients at other centers. There was also a gender bias with caregivers; despite actively trying to interview both parents, access was usually only given to the child’s mother. Telephone and face-to-face interviews both contributed to the knowledge gained in this study, the latter may have allowed a better rapport to be established with the interviewee, but only four telephone interviews were needed, despite the wide geographical spread of participants. Finally, the inclusion criteria for this study were designed to identify those patients with pHLH; however, at initial presentation it is difficult to distinguish between patients with pHLH and secondary HLH, and both require the same conventional therapy [11]; therefore, there is a possibility of misdiagnosis. Given the focus of the study on patients’ and caregivers’ perspectives there is a lack of clinical data provided by medical professionals, which limits the understanding of the impact of pHLH from a clinical perspective.

## Conclusions

Despite continuous optimization of pHLH management, survival appears to have plateaued over recent years [11]. The disease and its treatments continue to place a significant burden on the lives of patients and caregivers. This qualitative interview study adds to the overall understanding of the burden of pHLH and highlights the long-term effects of pHLH on the physical, emotional, and social well-being of patients and caregivers, and draws attention to their concerns. These results help to further the understanding and knowledge of pHLH within the medical community, which could aid diagnosis and promote the need for supportive services for patients and caregivers.

## Methods

### Study design and participant selection

This exploratory study was designed to evaluate the impact of HLH on the HRQoL of children and young adults requiring BMT and their parents/primary informal caregivers using a qualitative methodology rather than HRQoL instruments. The study was conducted in compliance with the institutional review board (IRB-approved) protocol [29], Good Clinical Practice, and applicable regulatory requirements. All data collected were considered strictly confidential in accordance with local laws and the requirements of any ethical review body.

Patients and their caregivers were identified through HLH patient advocacy/support groups in the USA and UK, who promoted the study. Initial contact was made either through a personal letter of invitation or a request via social media platforms, such as websites or Facebook. Any interested participants then contacted the research teams by email and prospective participants were assessed for eligibility by phone using a screening questionnaire. Due to the rarity of HLH and the qualitative nature of this study, recruitment continued until enrollment was exhausted (see “Thematic analysis” section).

Eligible participants had to be a parent or primary caregiver of a child (< 18 years of age) diagnosed with HLH that required BMT within the last 10 years (diagnosis reported by the child’s parent/caregiver), or a young adult (18–30 years of age) diagnosed with HLH that required BMT within the last 10 years (as reported by their parent/primary caregiver or self-reported). Participants were asked specifically about BMT to identify patients with pHLH. However, the more commonly used term HSCT, which includes BMT, will be used in this report. The main exclusion criteria were any psychiatric, cognitive, or other impairments that would interfere with an interview and ability to self-complete questionnaires. Eligible participants received all relevant study information and provided written informed consent in accordance with the International Conference on Harmonization GCP guideline E6. There were no restrictions on participants withdrawing from the study at any time.

### Assessments

Participant interviews comprised semi-structured questions; no PRO/HRQoL instruments were included. The interview guide was developed by two qualitative researchers (A. Nixon and D. Wild) who have conducted studies using this methodology across multiple indications and in healthy populations. The interview guide was reviewed by NovImmune SA and Dr Booth.

These questions were designed to understand the impact of HLH on the physical, emotional, and social well-being of both patient and caregivers, as well as any impact on relationships, including between family members or external relationships, such as in an educational setting. The structure of the questions was designed to assess important points in the patient journey, such as diagnosis, treatment, and life now.

### Interview procedure

Interviews were conducted either face-to-face or by telephone using a semi-structured interview guide. Two versions of the guide were used, one for young adult patients and one for the caregiver. The interview included various sections/themes: family overview; recall of diagnosis; experience/concerns during treatment and medical follow-up; how HLH affects quality of life, such as social/leisure activities, sleep, mood, emotions, and relationships; future with HLH (see Additional file 1: The guides). Each interview lasted approximately 1 h and was audio recorded and then transcribed for thematic analysis. Examples of participant quotes (full or partial quotes) are provided within the text and tables to reflect the impact of each theme on the caregivers and patients. Quotes are verbatim, but any sensitive information has been removed to ensure anonymity. Description has been included in brackets where needed for clarity.

### Thematic analysis

Thematic analysis was conducted according to Joffe and Yardley [30], where inductive coding was used to identify likely themes and categories in the data based on the interview guides. Deductive coding was then used to identify themes and categories emerging from within the data. Analysis was conducted by two experienced qualitative research analysts (A. Nixon [AN] and K. Moore [KM]) and facilitated by a qualitative software tool, MAXQDA (MAXQDA Version 12 was developed and distributed by VERBI Software based in Berlin, Germany). A final code book was developed iteratively, as described here, to provide consistency and quality control. This book was applied to several interviews initially and subsequently revised. These thematic codes (n = 87), which followed the themes, i.e. treatment descriptions and concerns, were applied to all subsequent interviews. To minimize the potential for bias, three interviews (12.5% of the interview total) were double coded by both analysts and reviewed to assess the application of themes and analytic techniques. In the event of a disagreement/misalignment of coding then a consensus was reached between AN and KM. There was no preselected sample size and the study continued until no further participants were enrolled; ‘data adequacy’, or the point at which no new information is obtained from additional qualitative data [31], was assessed using saturation tables [32].

## Supplementary Information

**Additional file 1.** Interview guides for young adults and caregivers.

## Data Availability

The datasets used and/or analyzed during the current study are available from the corresponding author on reasonable request. Anonymized datasets only can be made available to ensure participant confidentiality.

## Abbreviations

BMT: Bone marrow transplant
CNS: Central nervous system
HCP: Healthcare professionals
HLH: Hemophagocytic lymphohistiocytosis
HRQoL: Health-related quality of life
HSCT: Hematopoietic stem cell transplantation
IRB: Institutional review board
pHLH: Primary hemophagocytic lymphohistiocytosis
PRO: Patient-reported outcome
UK: United Kingdom
USA: United States of America

## References

[1] Astigarraga I, Gonzalez-Granado LI, Allende LM, Alsina L (2018). Haemophagocytic syndromes: the importance of early diagnosis and treatment. Pediatr (Barc)..

[2] Ishii E (2016). Hemophagocytic lymphohistiocytosis in children: pathogenesis and treatment. Front Pediatr.

[3] Risma KA, Marsh RA (2019). Hemophagocytic lymphohistiocytosis: clinical presentations and diagnosis. J Allergy Clin Immunol Pract.

[4] Seo JJ (2015). Hematopoietic cell transplantation for hemophagocytic lymphohistiocytosis: recent advances and controversies. Blood Res.

[5] George MR (2014). Hemophagocytic lymphohistiocytosis: review of etiologies and management. J Blood Med.

[6] Rosado FG, Kim AS (2013). Hemophagocytic lymphohistiocytosis: an update on diagnosis and pathogenesis. Am J Clin Pathol.

[7] Biank VF, Sheth MK, Talano J, Margolis D, Simpson P, Kugathasan S (2011). Association of Crohn's disease, thiopurines, and primary epstein-barr virus infection with hemophagocytic lymphohistiocytosis. J Pediatr.

[8] Niece JA, Rogers ZR, Ahmad N, Langevin AM, McClain KL (2010). Hemophagocytic lymphohistiocytosis in Texas: observations on ethnicity and race. Pediatr Blood Cancer.

[9] Janka GE (2012). Familial and acquired hemophagocytic lymphohistiocytosis. Annu Rev Med.

[10] Henter JI, Horne A, Aricó M, Egeler RM, Filipovich AH, Imashuku S (2007). HLH-2004: Diagnostic and therapeutic guidelines for hemophagocytic lymphohistiocytosis. Pediatr Blood Cancer.

[11] Bergsten E, Horne A, Aricó M, Astigarraga I, Egeler RM, Filipovich AH (2017). Confirmed efficacy of etoposide and dexamethasone in HLH treatment: long-term results of the cooperative HLH-2004 study. Blood.

[12] Trottestam H, Horne A, Aricò M, Egeler RM, Filipovich AH, Gadner H (2011). Chemoimmunotherapy for hemophagocytic lymphohistiocytosis: long-term results of the HLH-94 treatment protocol. Blood.

[13] Jordan MB (2018). Emergence of targeted therapy for hemophagocytic lymphohistiocytosis. The Hematologist..

[14] Hammarberg K, Kirkman M, de Lacey S (2016). Qualitative research methods: when to use them and how to judge them. Hum Reprod.

[15] O'Brien BC, Harris IB, Beckman TJ, Reed DA, Cook DA (2014). Standards for reporting qualitative research: a synthesis of recommendations. Acad Med.

[16] Oliver A, Greenberg CC (2009). Measuring outcomes in oncology treatment: the importance of patient-centered outcomes. Surg Clin N Am..

[17] Sen ES, Steward CG, Ramanan AV (2017). Diagnosing haemophagocytic syndrome. Arch Dis Child.

[18] Bloss S, Klemann C, Rother AK, Mehmecke S, Schumacher U, Mucke U (2017). Diagnostic needs for rare diseases and shared prediagnostic phenomena: results of a German-wide expert Delphi survey. PLoS ONE.

[19] Lin WC, Chen JS, Chiang MF, Hribar MR (2020). Applications of artificial intelligence to electronic health record data in ophthalmology. Transl Vis Sci Technol.

[20] Cancer Research UK. Etoposide (Etopophos, Vepesid) 2019. https://www.cancerresearchuk.org/about-cancer/cancer-in-general/treatment/cancer-drugs/drugs/etoposide.

[21] Thompson PA, Allen CE, Horton T, Jones JY, Vinks AA, McClain KL (2009). Severe neurologic side effects in patients being treated for hemophagocytic lymphohistiocytosis. Pediatr Blood Cancer.

[22] Aljebab F, Choonara I, Conroy S (2017). Systematic review of the toxicity of long-course oral corticosteroids in children. PLoS ONE.

[23] Li WHC, Chung JOK, Ho KY, Kwok BMC (2016). Play interventions to reduce anxiety and negative emotions in hospitalized children. BMC Pediatr.

[24] Pilapil M, Coletti DJ, Rabey C, DeLaet D (2017). Caring for the caregiver: supporting families of youth with special health care needs. Curr Probl Pediatr Adolesc Health Care.

[25] Edelstein H, Schippke J, Sheffe S, Kingsnorth S (2017). Children with medical complexity: a scoping review of interventions to support caregiver stress. Child Care Health Dev.

[26] Nafees B, Lloyd A, Dewilde S (2021). Estimating health state utilities in hemophagocytic lymphohistiocytosis. J Patient Rep Outcomes.

[27] Jackson J, Titman P, Butler S, Bond K, Rao A, Veys P, et al. Cognitive and psychosocial function post hematopoietic stem cell transplantation in children with hemophagocytic lymphohistiocytosis. J Allergy Clin Immunol. 2013;132(4):889–95.e1–3. 10.1016/j.jaci.2013.05.046 23987797

[28] Gelhorn HL, Tong S, McQuarrie K, Vernon C, Hanlon J, Maclaine G (2016). Patient-reported symptoms of tenosynovial giant cell tumors. Clin Ther.

[29] Salus Institutional Review Board (IRB). Protocol AL0001; Qualitative investigation into the impact of HLH on children and their informal caregivers. NovImmune SA; 2017.

[30] Joffe H, Yardley L, Marks D, Yardley L (2004). Content and thematic analysis. Research methods for clinical and health psychology.

[31] Morse JM (1995). The significance of saturation. Qual Health Res..

[32] Kerr C, Nixon A, Wild D (2010). Assessing and demonstrating data saturation in qualitative inquiry supporting patient-reported outcomes research. Expert Rev Pharmacoecon Outcomes Res.

