# Supplementary material for: A qualitative investigation into the impact of hemophagocytic lymphohistiocytosis on children and their caregivers
Source: Orphanet J Rare Dis. 2021 May 6;16:205. doi: 10.1186/s13023-021-01832-2 (PMC8101208; doi:10.1186/s13023-021-01832-2)
Supplement: Supplementary file 1 — Additional file 1. Interview guides for young adults and caregivers. [file 13023_2021_1832_MOESM1_ESM.docx]

# Additional file 1

**1. Interview guide (young adult version)**

**Introduction – face-to-face interview**

- Introduce yourself and anyone else present in the room
- Explain that you are working on behalf of Alacrita, an independent research company
- Provide a short overview of the study
  - The purpose of this study is to learn about the effect of HLH on patients and their families, how it affects different areas of their life and the lives of the parents or caregivers looking after a child or children with HLH
- Check they have read the Participant Information Sheet, and whether they have any questions
- Emphasize the following:
  - Confidentiality, including audio recording and any reports; no names will be included in any report
  - Their right not to answer individual questions you ask
  - Their right to leave the interview or study at any time
  - That there are no right or wrong answers; you want to hear their thoughts and opinions
  - The interview will be audio recorded so they need to speak clearly so that all their comments can be heard
  - As the interviewer, you are not a medical expert; your role is to ask questions and listen
  - They can take a break at any time during the interview
- Explain how the interview will work:
  - Sign the Informed Consent Form if haven’t already
  - Collect in Background Questionnaire if this hasn’t already been returned to the study team
  - Begin the audio recording
  - The interview will last no longer than one hour
- Any questions before starting?

**Introduction – telephone interview**

- Introduce yourself
- Explain that you are working on behalf of Alacrita, an independent research company
- Provide a short overview of the study
  - The purpose of this study is to learn about the effect of HLH on patients and their families, how it affects different areas of their life and the lives of their parents or caregivers looking after a child or children with HLH
- Check they have read the Participant Information Sheet, and whether they have any questions
- Emphasize the following:
  - Confidentiality, including audio recording and any reports; no names will be included in any reports
  - Their right not to answer individual questions you ask
  - Their right to stop the interview or leave the study at any time
  - That there are no right or wrong answers; you want to hear their thoughts and opinions
  - The interview will be audio recorded so they need to speak clearly so that all their comments can be heard
  - As the interviewer, you are not a medical expert; your role is to ask questions and listen
  - They can take a break at any time during the interview; the interview can be stopped and continued at another time or on another day if needed
- Provide an overview of how the interview will work:
  - Check that the Informed Consent Form has been signed and returned to the study team
  - Check that the Background Questionnaire has been returned to the study team
  - The interview will last no longer than one hour
- Any questions before starting?

**Part 1: Family overview (5 minutes)**

- Please tell me about your family
  - Who are the adults in your family?
  - Tell me about any brothers and sisters you have
    - How old are they?
    - Have they had HLH diagnosed?
  - Has anyone in your family died because of HLH?
  - Has anyone in your family died from an unknown disease either with or without a fever?

**Part 2: From diagnosis to treatment (15 mins)**

Listen and explore (as appropriate)

- What do you recall or have you been told about the time you were first unwell with HLH but it had not yet been diagnosed?
- What do you recall or have you been told about the time you were first diagnosed with HLH?
- What do you recall or have you been told about being treated for HLH?

**Part 3: Life now (20 mins)**

Listen and explore (as appropriate)

- Does having had HLH affect your life today?
  - Overall health
    1. Medical follow-up
    2. Medical treatment
    3. Medical prevention (e.g. infections)
  - Emotions
  - Mind or psychology
  - Sleep
  - Relationship with parents
  - Relationship with any brothers and/or sisters
  - Relationships with friends
  - Social activities
  - Leisure activities

**Part 4: The future (10 mins)**

Listen and explore (as appropriate)

- Do you feel that having had HLH has affected your future?

**Wrap-up**

- Ask if there are any questions before you finish the interview
- Thank the participant for their time and valuable contribution to the study
- Remind them that all the information shared will remain confidential, including audio recording and any reports; no names will be included in any report
- Explain that the study team will provide the £75/$100 voucher shortly following the interview either electronically via email or by post

### **2. Interview guide (caregiver version)**

**Introduction – face-to-face interview**

- Introduce yourself and anyone else present in the room
- Explain that you are working on behalf of Alacrita, an independent research company
- Provide a short overview of the study
  - The purpose of this study is to learn about the effect of HLH on patients and their families, how it affects different areas of their life and the lives of the parents or caregivers looking after a child or children with HLH
- Check they have read the Participant Information Sheet, and whether they have any questions
- Emphasize the following:
  - Confidentiality, including audio recording and any reports; no names will be included in any report
  - Their right not to answer individual questions you ask
  - Their right to leave the interview or study at any time
  - That there are no right or wrong answers; you want to hear their thoughts and opinions
  - The interview will be audio recorded so they need to speak clearly so that all their comments can be heard
  - As the interviewer, you are not a medical expert; your role is to ask questions and listen
  - They can take a break at any time during the interview
- Explain how the interview will work:
  - Sign the Informed Consent Form if haven’t already
  - Collect in Background Questionnaire if this hasn’t already been returned to the study team
  - Begin the audio recording
  - The interview will last no longer than one hour
- Any questions before starting?

**Introduction – telephone interview**

- Introduce yourself
- Explain that you are working on behalf of Alacrita, an independent research company
- Provide a short overview of the study
  - The purpose of this study is to learn about the effect of HLH on patients and their families, how it affects different areas of their life and the lives of the parents or caregivers looking after a child or children with HLH
- Check they have read the Participant Information Sheet, and whether they have any questions
- Emphasize the following:
  - Confidentiality, including audio recording and any reports; no names will be included in any reports
  - Their right not to answer individual questions you ask
  - Their right to stop the interview or leave the study at any time
  - That there are no right or wrong answers; you want to hear their thoughts and opinions
  - The interview will be audio recorded so they need to speak clearly so that all their comments can be heard
  - As the interviewer, you are not a medical expert; your role is to ask questions and listen
  - They can take a break at any time during the interview; the interview can be stopped and continued at another time or on another day if needed
- Provide an overview of how the interview will work:
  - Check that the Informed Consent Form has been signed and returned to the study team
  - Check that the Background Questionnaire has been returned to the study team
  - The interview will last no longer than one hour
- Any questions before starting?

**Part 1: Family overview (5 minutes)**

- Please tell me about your family
  - Who are the adults in your family?
  - Tell me about the children in your family
    - How old are they?
    - Have they had HLH diagnosed?
  - Has anyone in your family died because of HLH?

**Part 2: Period when child was first unwell but not yet diagnosed with HLH (15 minutes)**

Listen and explore (as appropriate)

- Impact on child/children
  - Typical day for your child during this time
  - Typical night for your child during this time
  - Child’s health
  - Child’s sleep
  - Child’s emotions
  - Effect on the child’s time at school or nursery
  - Any issues related to parents being more protective
  - Older children only
    - Child’s relationships with friends
    - Child’s social activities
    - Child’s relationship with their parents
    - Child’s relationship with any brothers and/or sisters
    - Effect on the child’s mind or psychology
    - Effect on the child’s leisure activities
- Impact on caregiver
  - A typical day for you during this time
  - A typical night for you during this time
  - Impact on your family
  - Impact on your relationships with people outside of the family
  - Impact on your work or study
  - Effect on your own health
  - Effect on your mind
  - Effect on your emotions
  - Did you feel other people were judging you during this time?
  - Effect on your social life
  - Effect on your leisure activities

**Part 3: Time child was first diagnosed with HLH (15 mins)**

Listen and explore (as appropriate)

- Impact on child/children
  - Typical day for your child during this time
  - Typical night for your child during this time
  - Child’s health
  - Child’s sleep
  - Child’s emotions
  - Effect on their child’s time at school or nursery
  - Any issues related to parents being more protective
  - Older children only
  - *Child’s relationships with friends*
  - *Child’s social activities*
  - *Child’s relationship with their parents*
  - *Child’s relationship with any brothers and/or sisters*
  - *Effect on the child’s mind or psychology*
  - *Effect on the child’s leisure activities*
- Impact on caregiver -make it clear that these questions are for the caregiver to answer about themselves unless otherwise stated
  - A typical day for you during this time
  - A typical night for you during this time
  - Impact on your family
  - Impact on relationships with people outside of the family
  - Impact on your work or study
  - Effect on your own health
  - Effect on your mind
  - Effect on your emotions
  - Did you feel that other people were judging you during this time?
  - Effect on your social life
  - Effect on your leisure activities

**Part 4: Period whilst child was being treated for HLH (15 mins)**

Listen and explore (as appropriate)

- Impact on child/children
  - Typical day for your child during this time
  - Typical night for your child during this time
  - Child’s health
  - Child’s sleep
  - Child’s emotions
  - Effect on their child’s time at school or nursery
  - Any issues related to parents being more protective
  - *Older children only*
    - *Child’s relationships with friends*
    - *Child’s social activities*
    - *Child’s relationship with their parents*
    - *Child’s relationship with any brothers and/or sisters*
    - *Effect on the child’s mind or psychology*
    - *Effect on the child’s leisure activities*
- Impact on caregiver
  - A typical day for you during this time
  - A typical night for you during this time
  - Any concerns related to the treatment
  - Impact on your family
  - Effect on your own health
  - Effect on your mind
  - Effect on your emotions
  - Did you feel that other people were judging you during this time?
  - Impact on relationships with people outside of the family
  - Impact on your work or study
  - Effect on your social life
  - Effect on your leisure activities

**Part 5: Life now (10 mins)**

Listen and explore (as appropriate)

- *Impact on child/children – only for HLH survivors*
  - Typical day for your child during this time
  - Typical night for your child during this time
  - Child’s health
  - Child’s sleep
  - Child’s emotions
  - Effect on their child’s time at school or nursery
  - Any issues related to parents being more protective
  - Any issues related to concerns around sterility
  - *Older children only*
    - *Child’s relationships with friends*
    - *Child’s social activities*
    - *Child’s relationship with their parents*
    - *Child’s relationship with any brothers and/or sisters*
    - *Effect on the child’s mind or psychology*
    - *Effect on the child’s leisure activities*
- Impact on caregiver
  - A typical day for you during this time
  - A typical night for you during this time
  - Impact on your family
  - Impact on relationships with people outside of the family
  - Impact on your work or study
  - Effect on your own health
  - Effect on your mind
  - Effect on your emotions
  - Did you feel other people were judging you during this time?
  - Effect on your social life
  - Effect on your leisure activities
  - *Only for HLH survivors*
    - Any issues related to concerns around your child’s future?

**Wrap-up**

- Ask if there are any questions before you finish the interview
- Thank the participant for their time and valuable contribution to the study
- Remind them that all the information shared will remain confidential, including audio recording and any reports; no names will be included in any report

Explain that the study team will provide the £75/$100 voucher shortly following the interview either electronically via email or by post.
